# Inhibitory effect of essential oils obtained from plants grown in Colombia on yellow fever virus replication *in vitro*

**DOI:** 10.1186/1476-0711-8-8

**Published:** 2009-03-06

**Authors:** Rocío Meneses, Raquel E Ocazionez, Jairo R Martínez, Elena E Stashenko

**Affiliations:** 1 Centro Nacional de Investigaciones para la Agroindustrialización de Especies Vegetales Aromáticas y Medicinales Tropicales, CENIVAM. Universidad Industrial de Santander, Bucaramanga, Colombia; 2 Centro de Investigaciones en Enfermedades Tropicales, CINTROP, Laboratorio de Arbovirus, Piedecuesta, Colombia; 3 Centro de Investigación en Biomoléculas, CIBIMOL, Bucaramanga, Colombia

## Abstract

**Background:**

An antiviral drug is needed for the treatment of patients suffering from yellow fever. Several compounds present in plants can inactive *in vitro *a wide spectrum of animal viruses.

**Aim:**

In the present study the inhibitory effect of essential oils of *Lippia alba, Lippia origanoides, Oreganum vulgare *and *Artemisia vulgaris *on yellow fever virus (YFV) replication was investigated.

**Methods:**

The cytotoxicity (CC_50_) on Vero cells was evaluated by the MTT reduction method. The minimum concentration of the essential oil that inhibited virus titer by more than 50% (MIC) was determined by virus yield reduction assay. YFV was incubated 24 h at 4°C with essential oil before adsorption on Vero cell, and viral replication was carried out in the absence or presence of essential oil. Vero cells were exposed to essential oil 24 h at 37°C before the adsorption of untreated-virus.

**Results:**

The CC_50 _values were less than 100 μg/mL and the MIC values were 3.7 and 11.1 μg/mL. The CC_50_/MIC ratio was of 22.9, 26.4, 26.5 and 8.8 for *L. alba, L origanoides, O. vulgare and A. vulgaris*, respectively. The presence of essential oil in the culture medium enhances the antiviral effect: *L. origanoides *oil at 11.1 μg/mLproduced a 100% reduction of virus yield, and the same result was observed with *L. alba, O. vulgare *and *A. vulgaris *oils at100 μg/mL. No reduction of virus yield was observed when Vero cells were treated with essential oil before the adsorption of untreated-virus.

**Conclusion:**

The essential oils evaluated in the study showed antiviral activities against YFV. The mode of action seems to be direct virus inactivation.

## Background

Yellow fever (YF) is a viral hemorrhagic fever endemic in South America and sub-Saharan Africa. It is caused by the yellow fever virus (YFV) that is transmitted to humans through the bite of the *Aedes *or *Haemagogus *mosquitoes [1]. YFV, belonging to the *Flavivirus *genus, is a single-stranded RNA genome virus that possess a spherical nucleo-capsid surrounded by a lipid envelop in which the envelope (E) protein and membrane (M) protein are embedded. Binding of YFV to the cell surface is believed to be mediated by the E protein [2].

Classical YF illness characterized by abrupt fever, myalgia, headache and conjunctival injection is a self-limiting disease. Few patients (15%) develop severe disease with jaundice, renal dysfunction, myocardial injury, and a bleeding diathesis. Within this group of patients, 20 to 50% end fatally [1]. The World Health Organization estimates that at least 200,000 cases of YF are reported each year, including 30,000 deaths [3]. This is despite that YF is prevented by vaccination with a live attenuated strain (17D) of the YFV [4]. Although the 17D vaccine has a long record of safety, in a few vaccinees (1:200,000 – 1:300,000) the virus can cause adverse events, such as meningoencephalitis or an acute viral hemorrhagic syndrome with multiple organ system failure. The fatality rate in cases suffering from disease due to 17D vaccination is 60% [4,5]. There is no currently approved antiviral drug against YF.

Numerous studies have been conducted exploring different approaches to antiviral compounds against YFV. These include, chemical compounds focused at virus replication interruption or at inhibition of a specific host pathway critical to virus replication without undue toxicity; and strategies aimed at modulation of host immune response using passive antibodies, immunomodulators and corticosteroids [6]. Although some of those approaches have shown promising results, any of them has yet to be approved for use in humans.

Natural products are an important source to develop antiviral drugs, and they are selected frequently on the basis of their ethno-medicinal use [7]. Several reports have demonstrated direct inactivation and prevention of cell-to-cell spread, of essential oils obtained from a varied number of plants on enveloped viruses that cause human disease. [8-14]. Nonetheless, little is known about antiviral effect on *Flavivirus*. To our knowledge, studies evaluating antiviral effect of essential oils against YFV have not been reported.

The objective of this study was to evaluate the inhibitory effect of essential oils obtained from *Lippia alba*, *Lippia origanoides*, *Origanum vulgare *and *Artemisia vulgaris *grown in Colombia on YFV replication *in vitro*. Plants are widely used all over the world to cure almost any disease. Infusions are recommended against liver diseases; digestive, spasmolitic and relieve stomach-ache; kidney pains; and respiratory disorders. It is speculated that some of the mentioned medicinal properties could be related to the plant's essential oil compounds [15,16].

## Methods

### Essential oil

Propagation cuttings were obtained from *L. alba *and *L. origanoides *growing wild in the surroundings of the Jordán Sube township (Department of Santander). The cuttings were used in establishing experimental contiguous plots in the CENIVAM Agroindustrial Pilot Complex, located at the main campus of Universidad Industrial de Santander (Bucaramanga, Colombia). *O. vulgare *and *A. vulgaris *were collected in Marinilla (Department of Antioquia) and Armenia (Department of Quindio), respectively. Voucher specimens from each plant were deposited at the Colombian National Herbarium (COL), and the taxonomic identifications were performed by Dr José Luis Fernández (National Herbarium, UN, Bogotá, Colombia).

For all oils, the dried plant material was submitted to microwave-assisted hydrodistillation in a Clevenger-type apparatus, as described elsewhere [17]. Compound identification was based on chromatographic (retention times, retention indices, standards) and spectroscopic (spectral interpretation, comparison with databases and standards) criteria [17,18]. Two GC-MS systems were employed, an Agilent Technologies 6890 Plus gas chromatograph (Palo Alto, CA, U.S.A.), equipped with an Agilent Technologies 5973N mass selective detector, and an Agilent Technologies 6890 gas chromatograph coupled to an Agilent Technologies 5975 mass selective detector. Both systems were equipped with a split/splitless injector (split ratio 1:50), a 7863 automatic injector and an MS-ChemStation G1701-DA data system that included the spectral libraries WILEY 138K, NIST 2002 and QUADLIB 2004. A fused silica capillary column DB-5MS (J&W Scientific, Folsom, CA, U.S.A.) of 60 m × 0.25 mm I.D. × 0.25 μm, d_f_, and a fused silica DB-WAX (J & W Scientific, Folsom, CA, U.S.A.) 60 m × 0.25 mm, D.I × 0.25 μm, d_f _column were employed. The oven temperature was programmed from 45°C (5 min) to 150°C (2 min) at 4°C min^-1^, then to 250°C (5 min) at 5°C min^-1^, and finally, to 275°C (15 min) at 10°C min^-1^. The ionization chamber and transfer line temperatures were kept at 230°C and 285°C, respectively.

Plant local names, voucher numbers and the main essential oil components are shown in Table 1. For the antiviral assays, the essential oil was dissolved in a solution of 1% dimethyl sulfoxide (DMSO) in test medium.

**Table 1:** Plants used in the study and their main essential oil components

Specie (voucher)	Local name	Main essential oil components (%)	Extraction yield (% p/p)
*Lippia alba *(CO 480750)*	Pronto alivio	Carvone (51)	0.4
		Limonene (33)	
		bicyclosesquiphellandrene (7)	
*Lippia origanoides *(CO 512075)*	Orégano de monte	Carvacrol (44)	3.5
		Thymol (15)	
		γ-terpinene (10)	
*Origanum vulgare *(CO 523701)^†^	Orégano	*trans*-Sabinene hydrate (21)	0.3
		Thymol (11)	
		Carvacryl methyl ether (11)	
		γ-Terpineno (5.2)	
		*p*-Cimene (4.5)	
*Artemisia vulgaris *(CO 517002)^†^	Ajenjo	α-Thujone (38.1)	0.1
		β-Thujone (10.6)	
		1,8-Cineole (8.8)	
		*trans*-Carveol (3.1)	
		Sabineno (2.8)	

Source: *, Ref. 17 and 18; †, this study.

### Cell culture and virus

African green monkey kidney cells (Vero) were maintained in minimum essential medium (M-199) supplemented with 10% fetal calf serum (FCS) and 0.07% NaHCO_3 _at 37°C in a humidified 5%-CO_2 _atmosphere. The vaccine strain 17D of YFV was provided by the state Secretariat of Health. Virus stocks were prepared by infecting Vero cell cultures; the supernatant was collected 2–3 days post infection and stored at -70°C in aliquots.

### Plaque assay

Virus titers were determined by plaque assays in Vero cells growing in 24-well plates. Briefly, serial tenfold dilutions of the viral suspension were added (0.1 mL/well) in duplicate, and the cells were incubated for 1 h at 37°C. Subsequently, M-199 medium containing 5% FCS and 3% carboxymethyl-cellulose (Sigma; 0.5 mL/well plates) was added, and the plate was incubated for 72 h at 37°C. The viral plaques were visualized after 10% formaldehyde fixation (1 h at room temperature) and by staining (15 to 30 s) with a 1% crystal violet solution. The titer was estimated by counting the number of plaques observed in each well and expressed as plaque-formation unit per milliliter (PFU/mL).

### Cytotoxicity

Cell viability was measured on the basis of the mitochondrial-dependent reduction of MTT to formazan [19]. Vero cells grown in a 96-well plate were treated with the essential oil at concentrations of 100, 33.3, 11.1 and 3.7 μg/mL for 72 h at 37°C. Then, the culture medium was removed and MTT solution (10 μL, 5 mg/mL, Sigma Co.) was added to each well. The plate was incubated for 4 h at 37°C and DMSO (100 μL) was added to the wells to dissolve the MTT crystals. Each essential oil was tested in triplicate. The extent of MTT reduction to formazan within the cells was quantified by measuring absorbance at 595 nm (OD_595_) on a multiwell spectrophotometer (Sensident Scan Merck). The 50% cytotoxic concentration (CC_50_) was defined as the concentration that reduces the OD_590 _of treated cells to 50% with respect to untreated cells. The CC_50 _values were calculated using the MSx/fitTM; ID Business Solution, Guildford, UK software.

### Viral inhibitory effect

The direct inactivation of YFV by the selected essential oils was tested by using a virus yield reduction assay. The virus (9.5 × 10^4 ^PFU) was incubated with concentrations of the essential oil (100, 33.3, 11.1 and 3.7 μg/mL) for 24 h at 4°C in M-199 medium with 2% FCS. Treated-YFV was used to infect monolayers of Vero cell grown in 24-well plates at a multiplicity of infection of 1 PFU per cell (MOI = 1). After a 1 h period at 37°C, the cells were washed twice and then incubated at 37°C in M-199 medium containing 5% FCS. Some experiments were also performed incubating the virus-infected cells in M-199 medium containing essential oil at concentrations described above. In all assays, the supernatant, consisting of culture medium, was collected and cleared by centrifugation at 400 × g at 4°C to determine the virus titer by the plaque assay method 48 h after incubation of the plates. The results were expressed as the lowest concentration of the essential oil at which reduction of virus titer by more than 50% (compared with control) was observed, and it was considered as the minimum inhibitory concentration (MIC).

The interference of the tested essential oils with the viral cycle by treatment of cells before viral adsorption was evaluated. Confluent monolayers of Vero cells grown in 24-well plates were exposed or not to the essential oil at concentrations described above for 24 h at 37°C. The cells were washed three times to remove residual oil, and then were infected with untrated YFV at MOI of 1 for 1 h at 37°C. The cells were washed three times to remove residual oil, and then were infected with untreated-YFV at MOI of 1 for 1 h at 37°C. After that, the unabsorbed virus were removed by cell washes and the cells were incubated in M-199 medium with 5% FCS at 37°C. At 48 h after incubation, the culture medium was collected and cleared by centrifugation at 400 × g at 4°C to determine the virus titer by the plaque assay method.

## Results

Essential oils were tested for their cytotoxicity on Vero cell prior to the determination of their inhibitory effect against YFV. All essential oils were not cytotoxic. The CC_50 _for *L. alba*, *L. origanoides, O. vulgare *and *A. vulgaris *were 90 ± 15, 98 ± 3.8, 98 ± 2.8 and 98 ± 2.9 μg/mL, respectively.

**Figure 1:**
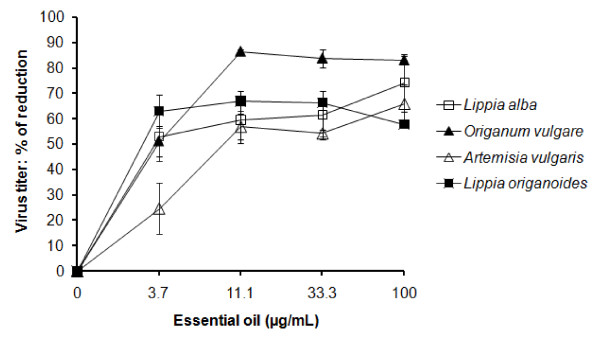
**Direct inactivation of essential oils from Colombian plants on yellow fever virus (YFV)**. About 9.5 × 10^4 ^PFU of YFV were incubated for 24 h at 4°C with concentrations of essential oil and then were adsorbed on Vero cells. Virus titers in supernatants of cell cultures were determined by plaque assays method at 48 h after adsorption. The data represent the means for three replicate samples of two independent experiment. Error bars indicate standard deviations.

The abilities of the selected essential oils to directly inactivate YFV (virucidal activities) were evaluated. Pre-incubation of virus with selected essential oil for 24 h at 4°C before adsorption on Vero cell inhibited the subsequent extracellular virus titer. The MIC for *L. alba*, *L origanoides *and *O. vulgare *oils *was *3.7 μg/mL whereas for *A. vulgaris *was 11.1 μg/mL (Figure 1). The MIC was lower than the CC_50 _for Vero cells, resulting a CC_50_/MIC ratio of 22.9, 26.4, 26.5 and 8.8 for *L. alba, L origanoides, O. vulgare and A. vulgaris*, respectively. A noted increased virucidal effect was observed with *O. vulgare *at concentrations higher than the MIC (> 3.7 μg/ml), but the same was not observed with the others essential oils. A poor (r = 0.72, linear regression) concentration-dependent reduction was observed with all essential oils.

**Figure 2:**
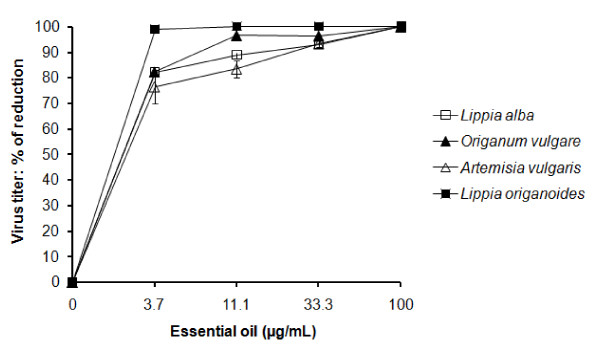
**Increase of direct yellow fever virus (YFV) inactivation by the presence of essential oil in the supernatant of virus-infected cells culture**. YFV previously incubated with serial dilutions of essential oil (Figure 1) was replicated in Vero cells at 37°C in M-199 medium containing varied concentration of essential oil. Virus titers in supernatants of cell cultures were determined by plaque assays method at 48 h after adsorption. The data represent the means for three replicate samples of two independent experiment. Error bars indicate standard deviations.

The presence of essential oil in culture medium for 48 h after the adsorption of treated-YFV on Vero cell monolayers enhances the antiviral effect (Figure 2). Virus titer was completely reduced in the presence of *L. origanoides *oil at concentration of 11.1 μg/mL, or of *L. alba, O. vulgare *or *A. vulgaris *oils at 100 μg/mL. This is, viral plaques were not recovered from undiluted media, and viral antigen was not detected on infected cells analyzed by microscopy-immunofluorescence.

**Figure 3:**
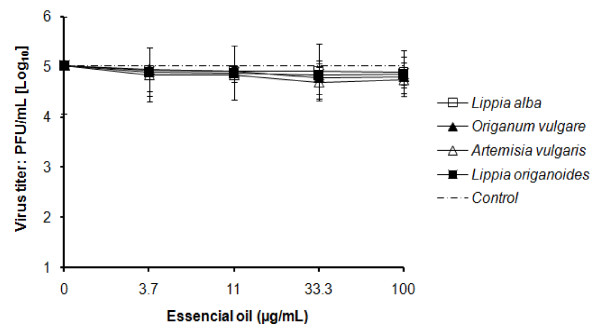
**Absence of antiviral effect of essential oils from Colombian plants on YFV replication by treatment of host cell**. Vero cells were exposed to varied concentration of essential oil for 24 h at 37°C before YFV infection (MOI = 1). Virus titers in supernatants of cell cultures were determined by plaque assays at 48 h after adsorption. The data represent the means for three replicate samples of two independent experiment. Error bars indicate standard deviations.

No inhibition was observed when cells were pre-incubated with essential oil for 24 h at 37°C before the adsorption of untreated-YFV. A significant reduction of viral progeny was not observed with none of the tested essential oils at concentration of 3.7, 11.1, 33.3 or 100 μg/mL (Figure 3). The virus titer in supernatants of cell cultures exposed to the essential oils did not show any difference with respect to the controls of untreated cells (p > 0.05, multiple regression).

Taken together, the results suggest that the action of essential oils as inhibitor agents of YFV infectivity is due to direct virus inactivation, preventing the adsorption and subsequent cellular infection.

## Discussion and conclusion

The antiviral or virucidal effect is generally expressed as IC_50 _(inhibitory concentration 50%). This is calculated by regression analyses of the dose response curve generated from the data. The IC_50 _value for all essential oils tested in this study could not be determined. This is because of concentrations lower than 3.7 or 11.1 μg/mL were not tested in order to investigate inhibition of virus titer by less than 50%. Consequently, we expressed virucidal activity as the lowest concentration of essential oil at which inhibition of virus titer by more than 50% was observed (MIC). On the other hand, definite essential oil concentration-dependent reduction was not observed (Figure 1). We could speculate that this result could be due to the strength of binding between virus and components of the essential oil which determines the fraction of molecules on the virus particle occupied by the oil. Even complete occupancy by using higher concentrations of the oil it could not be sufficient to increase the virus inhibition.

One method commonly used for evaluation of *in vitro *antiviral effect involves titration of residual virus infectivity after extracellular action of the test substance using a plaque reduction assay. Generally, this assay is run 1 h at 37°C or room temperature, and when the assay is performed at 4°C it is assumed that the inhibition may be lower [20]. Sadii et al. [10] found that the antiviral effect of essential oil obtained from *Artemisia arborescens *against enveloped Herpes Simplex virus type 1 was higher when the mixture virus-oil was incubated at 37°C than at 4°C. We evaluated the direct inactivation on YFV by essential oils from plants grown in Colombia in assays run at 4°C, using titration of residual virus in stored supernatant by using plaque assay method instead of direct reduction of viral plaque. Even with these limitations, the essential oils screened showed direct inactivation on YFV at MIC values as low as 3.7 and 11.1 μg/mL (Figure 2).

In this study, the direct YFV inactivation by selected essential oils was tested without any virus-oil mixture dilution to eliminate the oil. On the other hand, essential oil was added to culture medium after adsorption of treated-YFV. Consequently, the assays might not allow to precisely discriminate between antiviral and virucidal action because the oil could enter into the cells and interfere with intracellular steps of the viral cycle. As shown in Figure 3, we did not observe decrease of viral progeny when the cells were exposed 24 h to high concentration (100 μg/mL) of the essential oil and then were infected with untreated-YFV. This result suggests that changes on the plasmatic membrane cell or intracellular environment that could interfere with the adsorption or intracellular steps of the viral cycle were not induced. It was demonstrated that the presence of tested essential oils in culture medium during replication of treated-YFV enhances the inhibitory effect. This enhancement could be more probably due to direct inactivation of virus released from infected cells than inhibition of the penetration or protein synthesis steps of the viral cycle. Our results are in agreement with studies with other enveloped virus. The *in vitro *replication abilities of HSV [8-11,21-24], Human Immmodeficiency virus [12] and Hepatits B virus [13] were suppressed by previous exposition to essential oils obtained from plants from various countries, but antiviral activity was not observed by treatment of the cell with essential oil before the adsorption of the virus.

Supported studies of antiviral effect of essential oils on YFV were not found. Concerning antiviral activity on others members of the Flaviviridae family, two studies with dengue virus (DENV) have been reported. Duschatzky et al. [14] and Garcia et al. [25] did not observe significant antiviral effect on DENV-2 by essential oils obtained from eight species of aromatic plants from Argentina. Only *A. douglasiana *and *E. patens *had any discernible virucidal effect. We have carried out experiments for evaluation of direct inactivation on DENV-2 by essential oils from six plants grown in Colombia. *L. origanoides *and *Hyptis sp *showed a 50% reduction of the virus titer at concentration of 100 μg/mL, whereas *Piper auritum, Piper ottoniifolium, L. alba *and *Thymus vulgaris *did not show activity (unpublished data).

It is speculated that direct virus inactivation by the essential oils can be due to disruption of the viral membrane by lipophilic compounds [24], but the precise mechanism of the antiviral action is still not fully understood. The major components in the essential oil extracted from plants are terpenes and terpenoids. Several kinds of these compounds have been shown to inhibit HIV, and it was demonstrated that their anti-HIV activity involves inhibition of virus adsorption to target cell, and causing inactivation of HIV reverse transcriptase [26-29]. The major components of the essential oils tested in this study were carvone, carvacrol, limonene and thymol, and the presence of these compounds could in part explain the virucidal effect on YFV. More studies should be conducted to explore the mechanism of interference with viral intracellular process of the compounds present in the essential oils.

The present study has demonstrated the inhibitory effect of the essential oils obtained from *L. origanoides, O. vulgare, L. alba*, and *A. vulgaris *cultivated in Colombiaon YFV. The results obtained stimulate further investigation in order to know the antiviral activity of each compound present in the essential oil, and to determine whether or not synergism between them is responsible for that activity.

The topical use of essential oil might be explored for prevention of virus infection through the bite of the mosquitoes vectors.

## Competing interests

The authors declare that they have no competing interests.

## Authors' contributions

RML carried out the design of the study, performed the assays and edited of manuscript. REO conceived of the study, participated in it design and drafted the manuscript. JRM contributed to the identification of essential oils compounds. EES collected and analyzed the chemical data of essential oils.
